# Sexual Violence Suffered by Women in Early and Late Adolescence: Care Provided and Follow-Up

**DOI:** 10.1055/s-0042-1743094

**Published:** 2022-03-11

**Authors:** Alejandra Suyapa Becerra Torres, Ana Luiza Teixeira, Maria Teresa Ferreira Côrtes, Ândria Cléia Alves, Otávio Alabarse, Renata Cruz Soares de Azevedo, Arlete Fernandes

**Affiliations:** 1 Department of Gynecology and Obstetrics, Faculdade de Ciências Médicas, Universidade Estadual de Campinas, Campinas, SP, Brazil

**Keywords:** adolescence, women, sexual violence, sexual assault, mental health, emergency care, retrospective study, adolescência, mulher, violência sexual, agressão sexual, saúde mental, atendimento de emergência, estudo retrospectivo

## Abstract

**Objective** To compare the sexual violence suffered by women in early and late adolescence, the reactions triggered after the aggression, and the care provided.

**Methods** A retrospective study in which we reviewed the medical records of 521 female adolescents treated by a multidisciplinary team at a reference hospital in the city of Campinas, state of São Paulo, Brazil. We analyzed sociodemographic variables, and those pertainin to the characteristics of the episodes of violence, the emergency care, and the physical and psychological reactions observed during the follow-up. For the analysis, the sample was divided into groups of early (10 to 14 years) and late (15 to 18 years) adolescence. We used the Chi-squared/Fisher Exact, Mann-Whitney, and Kruskal-Wallis tests to compare the groups; the level of significance adopted was 5%.

**Results** The early group ( *n* = 242) contained more adolescents who were enrolled in school ( *p* < 0.001), suffered more daytime aggressions ( *p* = 0.031), in their residences ( *p* < 0.001), by an aggressor with whom they were acquainted ( *p* < 0.001), had greater need of legal protection ( *p* = 0.001), and took longer to seek care ( *p* = 0.048). Feelings of guilt, shame, and the perception of violence were similar between the groups. In the late group ( *n* = 279), there was greater consumption of alcohol during the aggression ( *p* = 0,005); they received significantly more prophylaxis treatments; reported more physical symptoms ( *p* = 0.033), sleep disorders ( *p* = 0.003), symptoms of anxiety ( *p* = 0.045), and feelings of anguish ( *p* = 0.011); and had more prescriptions of psychotropics ( *p* = 0.005). Only 52% completed the 6-month follow-up, with no differences between the groups.

**Conclusion** The age groups showed differences in the characteristics of the episodes of violence; early adolescents took longer to seek help, and the late group presented more intense symptoms and psychological worsening during the follow-up. Measures of prevention and specific care aimed at this population are needed.

## Resumo

**Objetivo** Comparar a violência sexual sofrida por vítimas no início e no final da adolescência, as reações desencadeadas após a agressão, e o cuidado de saúde dispensado.

**Métodos** Estudo retrospectivo, em que foram revisados os prontuários de 521 mulheres adolescentes atendidas por equipe multiprofissional em hospital de referência em Campinas, São Paulo, Brasil. As variáveis foram sociodemográficas, e aquelas relativas às características da violência, ao atendimento de emergência, e às reações físicas e psicológicas observadas durante o seguimento nos grupos de adolescentes de idade precoce (10a 14 anos) e tardia (15 a 18 anos). Utilizamos os testes do Qui-quadrado/Exato de Fisher, Mann-Whitney e Kruskal-Wallis para comparar os grupos; adotamos o nível de significância de 5%.

**Resultados** O grupo precoce ( *n* = 242) continha maior número de estudantes ( *p* < 0,001), que sofreram mais agressões diurnas ( *p* = 0,031), em suas residências ( *p* < 0,001), por agressor conhecido ( *p* < 0,001), tiveram maior necessidade de proteção legal ( *p* = 0,001), e demoraram mais a procurar atendimento ( *p* = 0,048). Sentimentos de culpa, vergonha e a percepção da violência foram similares entre os grupos. No grupo tardio ( *n* = 279) houve maior consumo de álcool durante a agressão ( *p* = 0,005); as adolescentes receberam significativamente mais tratamentos de profilaxia; relataram mais sintomas físicos ( *p* = 0,033), distúrbios do sono ( *p* = 0,003), sintomas de ansiedade ( *p* = 0,045), e sentimentos de angústia ( *p* = 0,011); e receberam mais prescrições de psicotrópicos ( *p* = 0,005). Apenas 52% completaram o seguimento de 6 meses, sem diferenças entre os grupos.

**Conclusão** Os grupos apresentaram diferenças nas características da violência; as adolescentes precoces chegaram mais tardiamente ao serviço, e o grupo tardio apresentou maior sintomatologia e piora psicológica no seguimento. São necessárias medidas de prevenção e cuidados específicos voltados a essa população.

## Introduction

Adolescence is the most vulnerable period for sexual violence due to the intrinsic changes in neurodevelopment 1 2 and some individual factors, including a history of domestic violence in childhood and risky behaviors such as the consumption of alcohol and/or other psychoactive substances (PASs). 3 4 5 6 7 Socioeconomic factors such as poverty, low level of schooling, and living in a society with high levels of violence can also influence and increase the vulnerability of adolescents. 5

There are ∼ 1.2 billion adolescents aged 10 to 19 years in the world. 8 According to statistical data collected in 190 countries, 9 ∼ 120 million girls have experienced forced sexual intercourse or other sexual acts at some point in their lives.

The consequences of sexual abuse to the health of women can be devastating and, their negative effects may be brief or last for a long time. In addition to physical injuries, the risk of unwanted pregnancy and sexually-transmitted infections (STIs), including by the human immunodeficiency virus (HIV), 5 as well as mental health disorders such as depression, anxiety, suicide, and posttraumatic stress, have also been described. 10 Although prophylactic treatment is effective to prevent and/or cure physical trauma, subsequent follow-up is essential to reduce the negative emotional responses, as well as the development of individual tools to deal with the trauma suffered and personal/family prejudices regarding sexual violence.

Studies 4 11 12 13 have shown differences in the characteristics of the episodes of sexual violence against the pediatric and adolescent populations. In Brazil, these differences have been shown in studies 14 15 16 based on national or regional reporting data. However, we do not know if there are differences in physical and emotional reactions, and in the expression of feelings triggered in the first months after sexual violence in adolescents of different ages.

The objectives of the present study were: to characterize the sexual violence suffered by adolescents in the age groups of 10 to 14 (early adolescence) and 15 to 18 years (late adolescence); to compare the physical, psychological and social reactions observed in the first six months of outpatient follow-up; and to characterize the medical care and social/legal demands of both groups.

## Methods

The present was a retrospective cohort study conducted at the Department of Gynecology and Obstetrics, School of Medical Sciences, Universidade Estadual de Campinas (UNICAMP), in the state of São Paulo, Brazil. The institutional Ethics Committee approved the project (under CAAE 20479819.4.0000.5404). We followed all items in the Strengthening of the Communication of Observational Studies in Epidemiology (STROBE) statement.

The study setting is a reference hospital in the city of Campinas and its metropolitan region, which covers a population of around 1.3 million inhabitants. It is a university hospital that provides emergency care to women who were victims of sexual violence, as well as a six-month outpatient follow-up conducted by a multidisciplinary team of gynecologists, psychiatrists, psychologists, nurses, and social workers.

We assessed adolescents aged 10 to 18 years who underwent emergency care from January 1st, 2011, to December 31st, 2018. We consulted the digitalized medical records of emergency care and outpatient follow-up performed by the multidisciplinary team. The last patient included had her medical records evaluated until July 1st, 2019, when the data collection was completed. Our service provides assistance regarding requests for the legal termination of pregnancy. The women are initially received by the social service professional and have an outpatient consultation scheduled for evaluation by the multiprofessional team. As this study aimed to evaluate the adolescents from of emergency care, it was not part of the scope to evaluate the adolescents who consulted with this condition.

The variables studied were: sociodemographic data (age, self-reported skin color, marital status, level of schooling, occupation, intellectual disability, and religion); type of abuse (acute: an event that is not repeated; and chronic: when the aggression is repeated over time and is perpetrated by the same aggressor/aggressors); the characteristics of the episodes of violence (time of occurrence, place of approach, form of intimidation, known aggressor and link with the aggressor, number of aggressors, type of violence, if the adolescent was under the influence of alcohol and/or other PASs at the time of the event, and whether there was “blackout” during the aggression); personal history (if the adolescent has started sexual activity, mental disorders, PASs addiction; affective disorders [depression, bipolarity; self-injurious behavior; eating disorders]; suicide attempts; need for psychotherapy or previous psychiatric treatment; intellectual disability; psychosis [psychotic disorder, schizophrenia, psychotic condition]; attention deficit hyperactivity disorder [ADHD]; anxiety disorder [panic attacks, phobias, generalized anxiety, obsessive compulsive disorder, adjustment disorder], previous sexual violence and age of occurrence); characteristics of the emergency care (time until emergency care was sought; prophylaxis performed [emergency contraception, prophylaxis against STIs, including HIV]; and collection of biological material for counterproof with the suspected offender); social needs/legal aid (shelters; contact with the Childhood Court of Justice and the Public Defender; protective measures; change of guardian; Guardian Committee notifications; and the calls for medical returns for which they did not attend); posttrauma reactions and changes referred/expressed during the follow-up (perception of violence; disclosure about the violence and which person she told; support received; physical disorders [sleep and appetite disturbances, physical disposition, gastrointestinal and urinary symptoms]); mental disorders (symptoms of anxiety and depression, suicidal ideations, suicide attempts, cutting, fears, flashbacks); social reactions (social isolation, changes in daily routine [irregular bedtime/wake-up and meal times; missed school/work/other activities; does not stay alone at home/does not go out alone], changes of address/city or changes of school); feelings expressed (apathy, anguish, crying, guilt, humiliation, insecurity, rage, shame) and psychotropic prescription during follow-up; and if a six-month follow-up with a multidisciplinary team was completed.

The psychologists who provided care defined the “perception of sexual violence” based on the reports of the adolescent reports, her mental organization and interpretation of the event, and by the experience lived as an act of sexual violence. “Feelings” are listed on the psychologists' clinical observation forms. All reactions and psychiatric symptoms were noted in medical records by the psychiatrists who cared for/followed up the adolescents.

For the comparison between the groups of women in early adolescence (10 to 14 years) and late adolescence (15 to 18 years), 17 we used the Chi-squared or Fisher exact tests for the categorical variables, and the Mann-Whitney and Kruskal-Wallis tests for the numerical variables. We used the Statistical Analysis System for Windows (SAS, SAS Institute Inc., Cary, NC, United States) software, version 9.2, and the level of significance adopted was 5%.

## Results

**Fig. 1:**
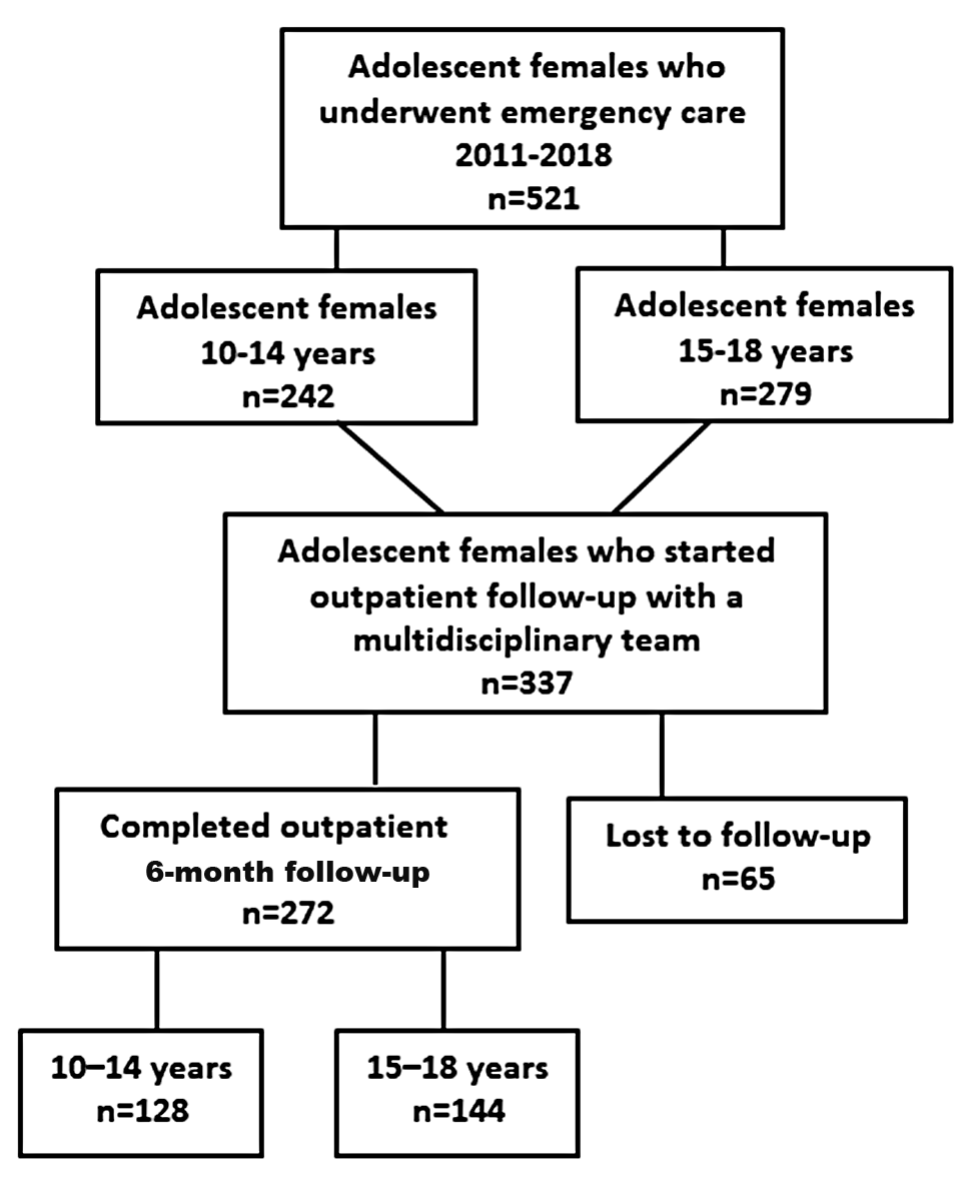
Flowchart of female adolescents who underwent emergency care after sexual violence, at an early age (10 to 14 years) and late (15 to 18 years) age, and who completed or were lost to the 6-month follow-up.

A total of 1,174 women received emergency care after sexual violence during the period of the present survey, 44.3% (521) of whom were adolescents aged 10 to 18 years (mean age: 14.8 ± 2.0 years) (data not shown). The sample was divided into 2 comparison groups consisting of 242 (46.5%) women in early adolescence and 279 (53.5%) in late adolescence ( Fig. 1 ).

Most adolescents were single (92%), students (80%), and 78% reported practicing some religion ( Table 1 ). About 8% of them lived with a partner, 14% were not enrolled in school (they were employed or without occupation), and 4.6% had some intellectual disability ( Table 1 ). The comparison between the groups showed significant differences in relation to occupation; the early group contained a higher number of students, and there were more adolescents employed or without occupation in the late group ( Table 1 ). Regarding personal history, there were more adolescents in the late group who had already started sexual life ( *p* < 0.001), who had a history of sexual violence ( *p* = 0.007), and had some kind of mental health disorder ( *p* = 0.008) ( Table 1 ). Among the 87 adolescents who reported a history of sexual violence, 70 referred to the age of occurrence of the event; of these, 54% reported the event occurred when they were between 4 and 10 years of age ( Table 1 ).

**Table 1:** Sociodemographic characteristics and personal history of female adolescents who were victims of sexual violence at the time of emergency care, according to age group

Sociodemographic characteristics and personal history	Age groups		*p* -value a
	10–14 years	15–18 years	
	n = 242	n = 279	
	**n (%)**	**n (%)**	
Self-reported skin color ( *n* = 517)			
White	145 (60.6)	187 (67.2)	0.119
Non-White	94 (39.3)	91 (32.7)	
Intellectual disability ( *n* = 521)			0.082
Yes	7 (2.9)	17 (6.1)	
No	235 (97.1)	262 (93.9)	
Years of schooling ( *n* = 517)			< 0.001
≤ 8	193 (80.1)	67 (24.3)	
> 8	48 (19.9)	209 (75.7)	
Marital status ( *n* = 521)			0.321
Single	226 (93.4)	254 (91.0)	
Cohabiting with partner	16 (6.6)	25 (8.9)	
Occupation ( *n* = 491)			< 0.001
Student	222 (97.3)	197 (74.9)	
Employed	1 (0.4)	30 (11.4)	
No occupation	5 (2.2)	36 (13.7)	
Religion ( *n* = 480)			0.448
Protestant	81 (37.5)	118 (44.7)	
Catholic	73 (33.8)	76 (28.8)	
Others	13 (6.0)	15 (5.7)	
Unaffiliated	49 (22.7)	55 (20.8)	
Sexual activity initiated ( *n* = 506)	44 (18.9)	108 (39.5)	< 0.001
History of sexual violence ( *n* = 511)	29 (12.2)	58 (21.2)	0.007
Age at first episode of violence ( *n* = 70)			0.585
4–10 years-old	13 (59.1)	25 (52.1)	
11–18 years-old	9 (40.9)	23 (47.9)	
History of mental disorders ( *n* = 428)	18 (8.6)	38 (17.2)	0.008

Note:

a Chi-squared test.

The characterization and context of the episodes of sexual violence are shown in Table 2 . Even with the high prevalence of acute abuse perpetrated by a single aggressor, we observed differences in the characteristics of the episodes of sexual violence between the groups. In the early group, a greater number of assaults occurred during the day ( *p* = 0.031), in their own residence or in those of acquaintances/family members ( *p* <0.001), and by a known abuser ( *p* <0.001) with a family or friendship bond ( *p* = 0.003); and a higher frequency of unsuccessful attempted sexual assaults was observed ( *p* = 0 *.008* ) when compared with the late group ( Table 2 ). The late group was more affected by nightly sexual assaults, perpetrated in public and in places routinely visited by the victims, or at parties, and the violence was perpetrated in greater number by unknown aggressors. Further, the late group experienced intimidation more often ( *p* = 0 *.* 002), as well as episodes of violence involving the use of a cutting weapon ( *p* = 0 *.* 008), and a higher frequency of oral intercourse ( *p* = 0 *.* 006). A greater number of the late adolescents did not know the type of violence suffered ( *p* = 0 *.* 036), were more often under the influence of alcohol ( *p* = 0 *.* 005), and reported that they had “blacked out” during the aggression ( *p* = 0 *.* 044) when compared with the early group. No other differences regarding the characteristics of the episodes of sexual violence were observed between the groups ( Table 2 ).

**Table 2:** Characteristics and context of the episode of sexual violence suffered by adolescent women according to age group

Characteristics and context of the episode of sexual violence	Age groups		*p* -value
	10–14 years	15–18 years	
	n (%)	n (%)	
Type of abuse			0.998 a
Acute	229 (94.6)	264 (94.6)	
Chronic	13 (5.3)	15 (5.4)	
Time of approach ( *n* = 512)			0.031 a
18:01–00:00 hours	82 (34.6)	94 (34.2)	
00:01–07:00 hours	50 (21.1)	84 (30.5)	
07:01–18:00 hours	105 (44.3)	97 (35.2)	
Place of approach ( *n* = 511)			< 0.001 b
Victim's residence	81 (34.4)	57 (20.6)	
Family residence	13 (5.5)	5 (1.8)	
Perpetrator's residence	12 (5.1)	10 (3.6)	
Acquaintance's residence	3 (1.28)	6 (2.1)	
Street	79 (33.6)	114 (41.3)	
Bus stop	5 (2.1)	13 (4.7)	
Other public places	3 (1.3)	11 (3.9)	
School	12 (5.1)	4 (1.4)	
Work	0	1 (0.3)	
Party	12 (5.1)	31 (11.2)	
Does not know	11 (4.7)	23 (8.3)	
Referred consent	4 (1.7)	1 (0.3)	
Known abuser ( *n* = 521)	154 (63.6)	126 (45.1)	< 0.001 a
Relationship with the aggressor ( *n* = 521)			0.003 a
Father	14 (5.8)	5 (1.8)	
Stepfather	14 (5.8)	8 (2.8)	
Other family member	16 (6.6)	12 (4.3)	
Friend	38 (15.7)	31 (11.1)	
School/work colleague	15 (6.2)	8 (2.8)	
Neighbor	9 (3.7)	10 (3.6)	
Intimate partner	8 (3.3)	10 (3.6)	
Other acquaintances	40 (16.5)	42 (15.0)	
Unknown	88 (36.3)	153 (54.8)	
Number of aggressors ( *n* = 516)			0.896 a
1	208 (87.0)	239 (86.3)	
2	21 (8.8)	24 (8.6)	
3–10	10 (4.2)	14 (5.0)	
Presence of intimidation ( *n* = 491)	188 (81.7)	230 (88.1)	0.002 a
Verbal threats ( *n* = 472)	37 (16.7)	58 (23.1)	0.085 a
Use of physical force ( *n* = 472)	120 (54.3)	132 (52.6)	0.710 a
Use of firearm ( *n* = 472)	12 (5.4)	20 (7.9)	0.274 a
Use of cutting weapon ( *n* = 472)	7 (3.1)	23 (9.1)	0.008 a
Forced inhalation ( *n* = 472)	9 (4.0)	7 (2.8)	0.452 a
Type of sexual violence ( *n* = 521)			
Vaginal aggression	156 (64.4)	175 (62.7)	0.681 a
Oral aggression	27 (11.6)	56 (20.0)	0.006 a
Anal aggression	34 (14.0)	42 (15.0)	0.746 a
Frustrated attempt	21 (8.7)	9 (3.2)	0.008 a
Undetermined aggression *	6 (2.5)	3 (1.1)	0.315 b
Does not know **	47 (19.4)	76 (27.2)	0.036 a
Alcohol consumption ( *n* = 469)	22 (9.8)	47 (19.1)	0.005 a
Consumption of other psychoactive substances ( *n* = 420)	6 (2.9)	11 (5.1)	0.247 a
“Blackout” during the aggression ( *n* = 515)	43 (18.0)	70 (25.3)	0.044 a

Notes:

a Chi-square test.

b Fisher's test.

* Undetermined aggression: the adolescent had memories of what happened, but could not distinguish the type of violence.

** Does not know: the adolescent was unable to answer because she couldn't remember what happened.

Table 3 shows their reactions and changes after the trauma. The perception of the experience as an act of sexual violence was highly prevalent in both groups. About two-thirds of the adolescents in both groups disclosed the episodes of sexual violence; the majority disclosed the event to parents or family members and reported having received support. There were no differences in these variables between the groups ( Table 3 ).

**Table 3:** Reactions, changes and feelings reported during the outpatient follow-up according to age group

Reactions and feelings after violence	Age groups		*p* -value
	10–14 years	15–18 years	
	n (%)	n (%)	
Perception of sexual violence ( *n* = 521)	210 (86.8)	255 (91.4)	0.301 a
Disclosure of the episode of sexual violence ( *n* = 414)	129 (65.8)	151 (69.2)	0.454 a
Person to whom the adolescent disclosed			
Mother ( *n* = 414)	48 (24.5)	48 (22.0)	0.552 a
Father ( *n* = 414)	17 (8.6)	22 (10.1)	0.622 a
Other relative ( *n* = 326)	15 (10.0)	13 (7.4)	0.401 a
Intimate partner ( *n* = 326)	4 (2.6)	12 (6.8)	0.084 a
Friend ( *n* = 414)	9 (4.6)	17 (7.8)	0.179 a
Other people ( *n* = 414)	4 (2.0)	12 (5.5)	0.068 a
Received support from someone ( *n* = 361)	123 (71.9)	145 (76.3)	0.341 a
Physical and/or psychosocial disorders ( *n* = 364)	155 (89.1)	173 (91.0)	0.529 a
Physical disorders ( *n* = 364)	73 (41.9)	101 (53.1)	0.033 a
Sleep disorders	58 (33.3)	93 (48.9)	0.003 a
Appetite disorders	37 (21.2)	44 (23.1)	0.664 a
Gastrointestinal disorders	6 (3.4)	8 (4.2)	0.706 a
Urogenital disorders	2 (1.1)	3 (1.6)	1.000 b
Changes in physical well-being	12 (6.9)	12 (6.3)	0.824 a
Mental Disorders ( *n* = 364)	106 (60.9)	132 (69.4)	0.087 a
Symptoms of anxiety	67 (38.5)	93 (48.9)	0.045 a
Symptoms of depression	25 (14.3)	39 (20.5)	0.123 a
Suicidal ideations	14 (8.0)	17 (8.9)	0.758 a
Suicide attempt	6 (3.4)	7 (3.7)	0.904 a
Cutting	5 (2.8)	2 (1.0)	0.266 b
Fear of the consequences of the episode of sexual violence	21 (12.0)	26 (13.7)	0.646 a
Fear of suffering sexual violence again	18 (10.3)	22 (11.6)	0.707 a
Flashbacks	24 (13.8)	41 (21.6)	0.053 a
Social changes ( *n* = 364)	135 (77.6)	160 (84.2)	0.107 a
Social isolation	38 (21.8)	55 (28.9)	0.120 a
Changes in daily routine	36 (20.7)	39 (20.5)	0.969 a
Changed address	14 (8.0)	17 (8.9)	0.758 a
Changed schools	13 (7.4)	4 (2.1)	0.015 a
Feelings expressed/perceived during health care ( *n* = 364)			
Shame	97 (55.7)	111 (58.4)	0.607 a
Guilt	77 (44.2)	82 (43.1)	0.833 a
Crying	14 (8.0)	23 (12.1)	0.201 a
Humiliation	8 (4.6)	16 (8.4)	0.142 a
Apathy	11 (6.3)	7 (3.7)	0.246 a
Anguish	2 (1.1)	12 (6.3)	0.011 a
Rage	4 (2.3)	5 (2.6)	1.000 b
Insecurity	3 (1.7)	8 (4.2)	0.166 a

Notes:

a Chi-squared test.

b Fisher exact test.

There were significant differences in the reactions after the episodes of violence and changes reported between the groups. The late group reported more physical symptoms ( *p* = 0.033), sleep disorders ( *p* = 0.003), symptoms of anxiety ( *p* = 0.045), and were prescribed more psychotropics, whereas those in the early group changed schools more often ( *p* = 0.015) ( Table 3 ). The feeling most expressed by both groups was shame, followed by guilt; only anguish was mentioned by a greater number of adolescents in the late group ( *p* = 0.011) ( Table 3 ).

Table 4 shows the care provided to the adolescents. Emergency care was provided up to 72 hours after the episode of violence to a greater number of adolescents in the late group ( *p* = 0.048), most of whom often received prophylaxis in the form of emergency contraception ( *p* = 0.003) and against STI and HIV ( *p* < 0.001), and had more biological samples collected ( *p* = 0.002) when compared with the early group ( Table 2 ). The early group required legal aid more often compared with the late group ( *p* = 0.001); only 53% of the families notified the police about the event, with no difference between the groups ( Table 4 ). Of the 521 adolescents admitted to the the emergency department, 337 (64.7%) started outpatient follow-up, and 272 completed the 6-month follow-up ( Fig. 1 ). The ages of the adolescents who completed the follow-up and of those lost to outpatient follow-up were similar, with a mean of 14.6 (standard deviation [SD]: ± 1.9) years and 14.7 (SD: ± 2.0) years respectively (data not shown).

**Table 4:** Emergency care and needs during the multidisciplinary follow-up according to age group

Characteristics of care	Age groups		*p* -value
	10–14 years	15–18 years	
	n (%)	n (%)	
Emergency care			
Time until search for medical care ( *n* = 514)			0.048 b
≤ 24 hours	108 (45.7)	154 (55.4)	
> 24–72 hours	44 (18.6)	54 (19.4)	
> 72 hours–5 days	19 (8.0)	23 (8.2)	
> 5 days until 6 months	63 (26.7)	44 (15.8)	
> 6 months	2 (0.8)	3 (1.0)	
Emergency contraception ( *n* = 462)	134 (62.9)	188 (75.5)	0.003 a
Prophylaxis against sexually-transmitted infections ( *n* = 511)	167 (75.5)	234 (87.3)	< 0.001 b
HIV prophylaxis ( *n* = 508)	135 (57.7)	197 (71.9)	< 0.001 a
Collection of biological sample ( *n* = 487)	98 (44.5)	156 (58.4)	0.002 a
*Six-month multidisciplinary follow-up*			
Assistance from social worker and/or legal aid ( *n* = 521)	181 (74.8)	172 (61.6)	0.001 a
Filing of police report ( *n* = 450)	112 (52.8)	127 (53.3)	0.910 a
Prescription of psychotropics ( *n* = 371)	42 (23.3)	70 (36.6)	0.005 a
Completed the 6-month follow-up *(n = 337)*	128 (79.0)	144 (82.3)	0.447 a

Notes:

a Chi-square test.

b Fisher exact test.

## Discussion

The prevalence of adolescents in relation to all women presenting to our service was similar to that of other studies, in which up to 50% of the reported cases of sexual violence occurred among adolescents. 9 12 13 16 In Brazil, sexual violence was the second most reported type of violence in the age group of 10 to 19 years, only exceeded by physical violence. 16 These rates show the impact of sexual violence on the health of women at a very young age, as well as the importance of ensuring access to emergency care for adolescents. In particular, there is a need to guarantee access to subsequent mental health disorders triggered by the sexual violence.

The fact that 1 out of 4 adolescents in the late group was not enrolled in school, added to the rate of 8% of adolescents living with a partner, raises the discussion about the adverse living conditions and the lack of conditions to complete formal education. These are situations that increase the vulnerability to sexual violence, and limit opportunities and personal development. Additionally, we observed 3.4% of intimate partner violence, a rate much lower than the 24% described in an analysis of health sector reports in Brazil from 2011 to 2017. 16 It is possible that the difference between the rates is related to the intrinsic characteristics of the population care for in our service. The prevalence of intellectual disability was higher than that of the general population, which has been reported to be of around 1%, corroborating the need for specific measures of protection, guidance, and care for these adolescents. 18

The largest number of adolescents who had initiated sexual life in the late group was expected and is in agreement with a national population-based study 19 that described a mean age of 15.3 years at first sexual intercourse among female adolescents aged between 16 and 19 years. On the other hand, the large number of adolescents with a history of child sexual abuse (CSA), 17% in the general sample and significantly higher in the late group, drew attention. Studies with adolescents 20 21 suggest that those exposed to CSA have greater sexual vulnerability during adolescence. In addition, the experience of sexual violence in childhood or adolescence can increase vulnerability to new episodes of abuse and the development of risky behaviors. 6 13 22 23 History of mental disorder was more frequently reported in the late group, which was expected. Difficulties in adapting to the transition to adulthood usually require some specific intervention.

The differences found in the characteristics of the episodes of violence between the groups can be explained by the greater exposure to social life and to experiences of new behaviors in late adolescence, while among the early group, the fact that they tend to remain closer to familiar environments, such as home and school, facilitates their exposure to aggressors who are their relatives or acquaintances. These differences are known and have been described in different studies 4 11 12 13 16 with children and adolescents, and are shown in the number of reports in Brazil.

Adolescents in the late group self-reported being under the influence of alcohol more often, as well as “blackouts” during the aggression due to the ingestion of alcohol and not knowing how to describe the aggression suffered; however, the rate of consumption was lower than the rates of 40% to 60% of consumption of alcohol and other PASs reported in studies 3 4 with victims of sexual violence. Despite the scarcity of national data, a recent article 24 reinforced this relationship. It is possible that the lower prevalence of PASs use may be related to the younger age of the women in the present study and because the information was self-reported, and no toxicological analyses were performed. Recent studies 25 26 have described drug-facilitated sexual assault (DFSA), a form of sexual violence against an individual incapacitated by a mind-altering substance such as alcohol or “rape drugs.” These drugs, when used in association with alcohol, can result in loss of consciousness and inability to consent to sexual intercourse. It is difficult to estimate the yearly number of DFSAs, considering the low report rates. Victims are often reluctant to report incidents out of embarrassment, because they feel judged, or because they do not clearly remember the attack.

At the time of the event, one in seven adolescents in the present study reported being under the influence of alcoholic beverages. This result reinforces the need to develop policies that encourage the system of attention to DFSA suspects in health services to collect detailed information about the individual's history with legal and illegal drugs. Adequate and more complete contextual information will serve to improve the quality of the care.

The late group was more symptomatic, with sleep disturbances, symptoms of anxiety, feelings of anguish, and psychotropic drugs were prescribed to 36% of them. This result shows the greater need for psychotherapy or mental health support in this period after trauma. A Brazilian study 27 performed with students at the end of Elementary School reported that students with a history of sexual abuse in childhood/early adolescence already showed an impact on some indicators of mental health, such as insomnia, a decrease in or absence of friends, and feelings of loneliness.

The perception of sexual violence was one of the reactions observed, and, 9 out of 10 adolescents evaluated realized they had suffered violence. This result corroborates the experience of more serious violence, such as perpetration by multiple aggressors and aggression by anal intercourse, experienced by one in seven victims, in addition to the fact that two-thirds of the adolescents had had their first sexual intercourse through aggression. These characteristics have been associated with worse emotional repercussions, which can hinder the recovery process of the adolescents. 9

During the outpatient follow-up, most adolescents described some type of physical, psychological and/or social reaction triggered by the aggression. We found a similar frequency regarding the feelings most mentioned by the two groups, guilt and shame, which surprised us. Although these feelings are often recognized after sexual violence in adult women, we believed that the younger group could be immersed in a less-prejudiced sociocultural environment. The “culture of rape” that sustains the victim's accountability, globally and in Brazil, promotes feelings of shame and guilt in victims. Unfortunately, we note that this representation is still very strong, even among the youngest; cultural and family values may influence the triggering of these feelings. These data indicate the relevance of evaluating this thematic in a future prospective study and the need for strategies to deal with this deleterious scenario.

In the present study, one in three adolescents did not disclose the experience to or did not feel supported by their family. Studies 28 have indicated that adolescents and children may never tell others about the aggression suffered or delay its disclosure for a long time, either because of fear, threats, lack of opportunity, or the nature of their relationship with the aggressor. This delay may have influenced the search for emergency care up to 72 hours after the incident for 35% of the early group and for 25% of the late group, which compromised the use of prophylaxis. The late search for care may also have been influenced by the lack of information about the service, the belief in the little importance of emergency care, or the difficulty in accessing the most peripheral regions of the city. A Brazilian study 29 analyzed data from 489 victims aged between 0 and 15 years, 369 of them female, reported by a pediatric hospital of reference in the city of Florianópolis, state of Santa Catarina, from 2008 to 2014, and included care data at the time of the report. Among girls, the most affected age group was between the ages of 10 and 15 years, and more than 70% of the victims did not undergo prophylactic procedures and material collection. The low rate of these procedures was attributed by the authors 29 to the non-application of the recommended protocol in cases of suspicion alone, without confirmation, to situations in which there was no indication for its application, and to the fact that care occurred after 72 hours. There is a lack of studies on the treatment/follow-up of adolescents who are victims of sexual violence in Brazil.

The early group required more the assistance of social workers or legal aid, a result that we attribute to the younger age of the victims, who often need protective measures. These results draw attention to the high vulnerability of this population, which largely depends on public policies and government protection.

Although two thirds of the adolescents started the six-month outpatient follow-up, just over half completed it, despite the measures taken to increase adherence. Our service routinely performs three calls for teenage victims who are not monitored by a multidisciplinary team, when necessary, with the assistance of the Guardian Committee.

The limitations of the present study include its retrospective design, which may have induced some bias in the results, mainly due to omissions in inserting information into the medical records. Another limitation was the low frequency of PAS consumption at the time of the aggression, when compared with reports in the literature. This result may be related to the difficulty of the victim to report this piece of information, and to the fact that we do not routinely carry out clinical tests to investigate the presence of drugs. Moreover, the possible diversity of professionals who provided care to the adolescents and the absence of a previous definition of “feelings,” forming a subjective basis for classification, may also have contributed to a bias in the results. On the other hand, the strength of the present study is that it enabled us to get to know the reactions caused after the trauma of sexual violence in both age groups of female adolescents. Due to the considerable number of adolescents followed by the multidisciplinary team, it was possible to compare the needs for change in routine life, the physical and emotional reactions, and feelings presented by adolescents after experiencing sexual violence in early and late adolescence. We did not find comparative studies on the subject. Sexual violence experienced during adolescence should be better analyzed in prospective studies.

It is important to highlight that, in the present study, psychological changes were described by 65.4% of the adolescents, which shows the impact of violence on mental health, and that medical services must be prepared to provide a multidisciplinary team, with nurses, gynecologists, social workers, and a mental health team for the follow-up aimed to minimize consequences. There is a need to improve the quality of information on the prevention and identification of cases of sexual violence against female adolescents and girls aimed at family members, caregivers and society, to reduce the time until the search for health care and the potential damage to the mental and sexual health of the adolescents. Our services must train professionals with sensitivity regarding the care of girls and adolescents who experience a situation of sexual violence.

## Conclusion

Universal health coverage for adolescents has been proposed to public policy managers as one of the paths to the sustainable development of nations. The Brazilian Unified Health Service (Sistema Único de Saúde, SUS, in Portuguese) provides universal coverage, that is, it provides care for the entire population; however, public policies for prevention, treatment and health education aimed at women's sexual and reproductive rights are very much needed.
